# Expression of Acid-Sensing Ion Channel 3 in Afferents Averts Long-Term Sensitization and the Development of Visceral Pain

**DOI:** 10.3390/ijms252312503

**Published:** 2024-11-21

**Authors:** Nicolas Montalbetti, Guadalupe Manrique-Maldonado, Youko Ikeda, Marianela Dalghi, Anthony Kanai, Gerard Apodaca, Marcelo D. Carattino

**Affiliations:** 1 Renal-Electrolyte Division, Department of Medicine, University of Pittsburgh, Pittsburg, PA 15261, USA; nim71@pitt.edu (N.M.); gum28@pitt.edu (G.M.-M.); yoi4@pitt.edu (Y.I.); mgd29@pitt.edu (M.D.); ajk5@pitt.edu (A.K.); gla6@pitt.edu (G.A.); 2 Department of Pharmacology and Chemical Biology, University of Pittsburgh, Pittsburg, PA 15261, USA; 3 Department of Cell Biology, University of Pittsburgh, Pittsburg, PA 15261, USA

**Keywords:** acid-sensing ion channels, urinary bladder, sensory neurons, chemical cystitis, visceral pain

## Abstract

Sensitization of primary afferents is essential for the development of pain, but the molecular events involved in this process and its reversal are poorly defined. Recent studies revealed that acid-sensing ion channels (ASICs) control the excitability of nociceptors in the urinary bladder. Using genetic and pharmacological tools we show that ASICs are functionally coupled with voltage-gated Ca^2+^ channels to mediate Ca^2+^ transients evoked by acidification in sensory neurons. Genetic deletion of *Asic3* of these sensory neurons does not alter the mechanical response of bladder afferents to distension in naïve mice. Both control and sensory neuron conditional *Asic3* knockout (*Asic3*-KO) mice with chemical cystitis induced by cyclophosphamide (CYP) administration exhibit frequent low volume voiding events. However, these changes are transient and revert over time. Of major significance, in *Asic3*-KO mice, CYP treatment results in the sensitization of a subset of bladder afferents and pelvic allodynia that persist beyond the resolution of the inflammatory process. Thus, ASICs function is necessary to prevent long-term sensitization of visceral nociceptors.

## 1. Introduction

The afferents that innervate the urinary bladder normally function to sense filling and promote voiding. However, in response to non-physiological excess filling, tissue insult, or injury, these afferents become sensitized exhibiting a wide range of molecular changes that alter their function and response to stimuli [1,2,3]. This persistent afferent drive increases the excitability of spinal dorsal horn neurons, a process referred as to “central sensitization”, which is responsible for the hypersensitivity to normally innocuous stimuli and expanded receptive field seen in acute and chronic visceral pain conditions [2,3,4,5,6,7]. Despite important advances in understanding the circuitry that controls urine storage and voiding [8,9,10,11,12], critical knowledge gaps remain about the molecular machinery that allows afferents to sense bladder distension and respond to pathologic events in a measured manner. The latter is important because an exacerbated response to injury or inflammation may lead to long-lasting pain that persists beyond the resolution of the pathologic process.

Ion channels in sensory neurons that responded to extracellular acidification were first described more than four decades ago by Krishtal and Pidoplichko [13]. Almost two decades after this initial report, a proton-gated cation channel that produced currents that resembled those observed in naïve neurons was cloned based on its homology with the epithelial sodium channel (ENaC) [14]. Five additional ASIC subunits and spliced variants have since been identified in rodents (ASIC1a, ASIC1b, ASIC2b, ASIC3 and ASIC4) [14,15,16,17,18,19,20,21,22]. ASICs subunits can associate to form proton-gated homo- and hetero-channels with unique functional properties [23]. An exception is ASIC4, which neither forms functional channels, nor does it contribute to functional heteromeric channels [21]. While ASICs were initially thought to function as mechanotransducers based on their homology to the *C. elegans* proteins *Mec4* and *Mec10*, two related members from the ENaC/Degenerin family; physiological studies carried out with global subunit null mice provided contradictory results (for extended discussion in this issue see [24]). In certain settings, genetic deletion of individual ASIC subunits enhanced mechanical sensitivity or nociceptive responses while in others reduced them. ASIC subunits are found in the neurons of the peripheral and central nervous systems, but their distribution within neuronal populations is complex and has not been fully resolved. The explanation for these conflicting results may be rooted in the ability of ASIC subunits to oligomerize and form functional channels on their own. For instance, in mouse skeletal muscle afferents, functional channels are formed by ASIC1, ASIC2 and ASIC3 [25]. However, genetic deletion of individual subunits does not eliminate proton-gated currents in neurons, but alters their biophysical properties including response to acidic solutions [25]. Additional uncertainty in the interpretation of whole animal studies arises from the broad distribution of ASIC subunits in both the periphery and the central nervous system, including areas that process incoming signals from the body and the environment (e.g., brainstem). To investigate the function of ASICs specifically in bladder afferents we used genetic tools including sensory neuron conditional *Asic3* knockout (*Asic3*-KO) mice, live-cell imaging and electrophysiological techniques.

## 2. Results

### 2.1. Genetic Deletion of Asic3 from Sensory Neurons Alters Proton Signaling

**Figure 1:**
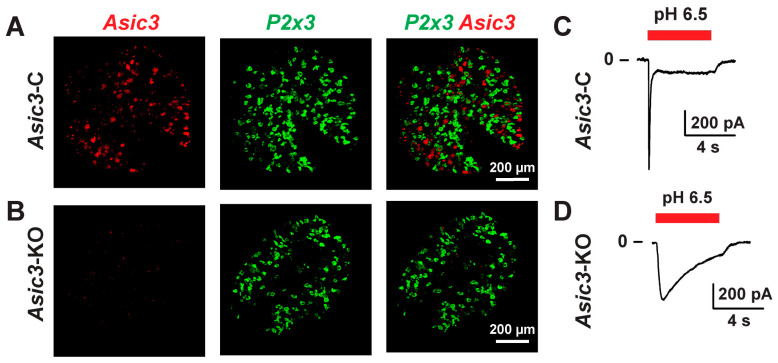
Genetic deletion of *Asic3* alters proton-evoked currents in sensory neurons. (**A**,**B**), *Avil*-induced Cre recombination disrupts *Asic3* expression in sensory neurons. Confocal images captured from cryosections of dorsal root ganglia (DRG) from control (*Asic3*-C; *Asic3^fl/fl^*) (**A**) and sensory neuron conditional *Asic3*-KO (*Avil*-Cre^+/−^; *Asic3^fl/fl^*) (**B**) mice. Fluorescence in situ hybridization (FISH) was performed with probes for *Asic3* and *P2rx3*. Data are representative of 3 experiments. (**C**,**D**), Genetic deletion of *Asic3* from sensory neurons alters the kinetics of proton-evoked currents. DRG were harvested from *Asic3*-C or *Asic3*-KO mice 7–10 days after injection of DiI in the bladder wall. Sensory neurons were isolated and cultured as indicated in Materials and Methods. Whole-cell currents were evoked by a change in extracellular pH from 8.0 to 6.5 (red line). Representative current tracings of ASIC-like currents from neurons of *Asic3*-C (**C**) and *Asic3*-KO (**D**) are shown (*n* = 5–9).

Functional acid-sensing ion channels are found in roughly 25% of the bladder sensory neurons [26]. Genetic deletion of the *Asic3* subunit eliminates the ability of bladder sensory neurons to generate action potentials in response to extracellular acidification [26,27], producing a functional ASIC knockout. Here, to delete *Asic3* from sensory neurons, we used a transgenic line that expresses Cre recombinase driven by the advillin (*Avil*) gene promoter in a constitutive manner (*Avil*-Cre) [28]. Note that *Avil* is almost exclusively expressed in sensory neurons [29], but not in other neuronal tissues apart from Mo5 motoneurons [29]. To confirm that *Avil*-driven Cre-mediated recombination eliminates *Asic3* from sensory neurons we used fluorescence in situ hybridization (FISH). As shown in Figure 1, *Asic3* expression is absent in sensory neurons of conditional *Asic3*-KO (*Asic3^fl/fl^*; *Avil*-Cre^+/−^) mice. No obvious changes in *P2x3* expression in sensory neurons were observed between control (*Asic3*-C, *Asic3^fl/fl^*) and conditional *Asic3*-KO mice. Additionally, we performed patch-clamp studies to confirm that the genetic deletion of *Asic3* alters ASIC function in sensory neurons. Consistent with previous reports [26,27,30,31], conditional deletion of *Asic3* slowed the desensitization of the proton-activated currents in sensory neurons (Figure 1C,D).

### 2.2. ASICs Are Functionally Coupled to Voltage-Gated Calcium Channels (VGCCs)

**Figure 2:**
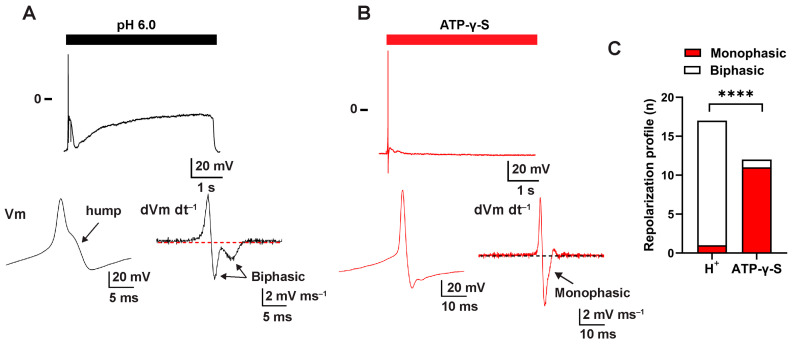
Protons evoke action potentials with repolarizing hump in sensory neurons. Sensory neurons were isolated and cultured as indicated in Section 4 Materials and Methods. The response to extracellular acidification (pH 6.0) or ATP-γ-S was evaluated in the voltage-clamp configuration of patch-clamp. Neurons that displayed ASIC-like or ATP-γ-S-gated currents were further studied in the current-clamp mode. (**A**,**B**), Representative tracing of action potential evoked by extracellular acidification (**A**) (indicated by the black line) or ATP-γ-S (**B**)(indicated by the red line). The lower left panels show an expanded time scale for action potentials evoked by extracellular acidification (pH 6.0) (**A**) or ATP-γ-S (10 μM) (**B**). The lower right traces are differentiated action potentials shown in (**A**,**B**). Note that action potentials evoked by extracellular acidification exhibit a characteristic hump during the repolarization phase, rending a biphasic waveform upon differentiation. (**C**), Relative incidence of neurons that respond to extracellular acidification or ATP-γ-S exhibiting a monophasic (red bars) or biphasic (open bars) differentiated waveform. Chi-square test, quadruple asterisks indicate a statistical difference, *p* < 0.0001, *n* = 12–17.

To understand how ASIC activation affects the sensory neuron function, we compared the repolarization profile of action potentials evoked by acidification or ATP-γ-S. The latter, a P2X agonist, was selected because it is known to produce generator potentials in bladder sensory neurons [32,33,34]. Visual inspection of action potentials evoked by acidification from previously published data reflects a characteristic “hump” (Figure 2A) that renders a biphasic shape upon differentiation (16/17 neurons) (Figure 2C). Note that while the properties of action potential evoked by acidification in this data set were previously reported [26], the repolarization profile analysis is new and has not been published previously. This hump is typically seen upon activation of VGCCs in dorsal and trigeminal sensory neurons [35,36] and it has been previously reported as a distinctive feature of the action potentials of nociceptive afferents [37]. In contrast, only 1 out of 12 action potentials evoked by ATP-γ-S exhibit a biphasic repolarization profile (Figure 2B,C). These results suggest functional coupling between ASICs and VGCCs in sensory neurons.

**Figure 3:**
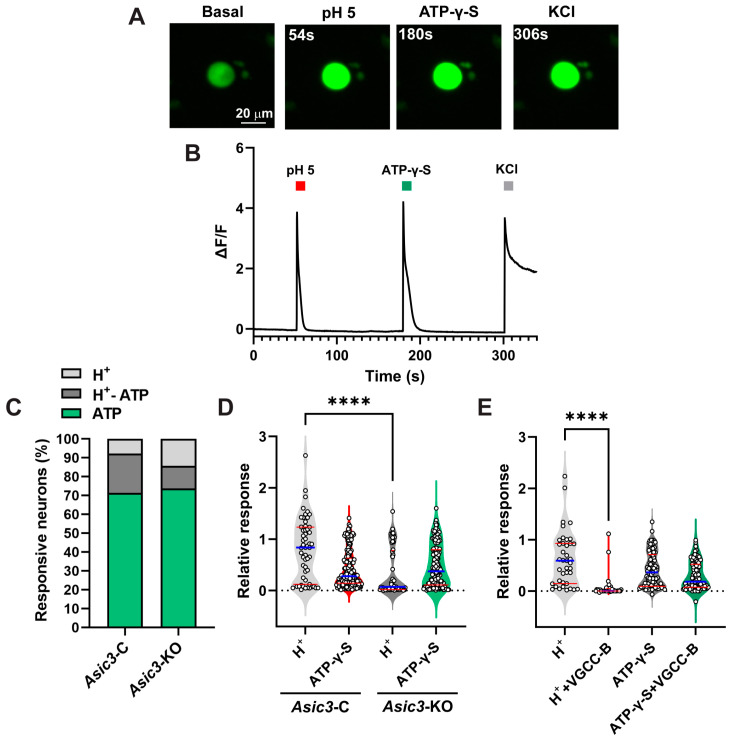
ASICs are functionally coupled to voltage-gated Ca^2+^ channels in sensory neurons. Dorsal root ganglia (DRG) were isolated from control (GCaMP5G^+/−^; *Avil*-Cre^+/−^) and conditional *Asic3*-KO (*Asic3^fl/fl^*;GCaMP5G^+/−^; *Avil*-Cre^+/−^) mice expressing GCaMP5G in sensory neurons. Ca^2+^ transients were examined in cultured sensory neurons 24–72 h after isolation. (**A**), Representative images showing proton and ATP-γ-S evoked Ca^2+^ transients in sensory neurons from control mice. (**B**), Representative tracing showing the change in fluorescence (ΔF/F) overtime for a sensory neuron from a control mouse. Sensory neurons were exposed to an extracellular acidic solution (red line), ATP-γ-S (green line) and lastly to a high K^+^ (KCl) solution (gray line) (see Section 4 Materials and Methods). (**C**), Percentage of sensory neurons that respond to extracellular acidification, ATP-γ-S, or both (*n* = 192–210). (**D**), *Asic3* is required for proton-evoked [Ca^2+^]_i_ transients in sensory neurons. Data, normalized to the Ca^2+^ transient evoked by KCl, are shown as the mean ± SEM (*n* = 55–178). Data were analyzed using Kruskal–Wallis test followed by Dunn’s multiple comparison test and a significant difference is indicated with a quadruple asterisks (*p* ≤ 0.0001). (**E**), Proton-evoked Ca^2+^ transients are mediated by voltage-gated Ca^2+^ channels (VGCCs) in sensory neurons. Neurons were exposed to an acidic solution or a solution containing ATP-γ-S (10 μM) in the absence or presence of VGCC blockers (VGCC-B; ω-conotoxin MVIIC, 100 nM; mibefradil, 10 μM; nifedipine, 10 μM). Data, normalized to the Ca^2+^ transient evoked by KCl, are shown as the mean ± SEM (*n* = 39–91). Data were analyzed using Kruskal–Wallis test followed by Dunn’s multiple comparison test and a significant difference is indicated with a quadruple asterisk (*p* ≤ 0.0001).

To explore the possibility of ASIC/VGCC coupling, we performed calcium imaging with the fluorescent Ca^2+^ sensor GCaMP5G in bladder sensory neurons from control (GCaMP5G^+/−^; *Avil*-Cre^+/−^) and sensory neuron conditional *Asic3*-KO (*Asic3^fl/fl^*; GCaMP5G^+/−^; *Avil*-Cre^+/−^) mice. Note that we use the *Avil*-Cre line for both genetic deletion of *Asic3* and expression of GCaMP5G. To determine if ASIC activation induces an intracellular Ca^2+^ transient in sensory neurons, DRG neurons were exposed to a drop in extracellular pH from 7.4 to 5.0, followed by 10 μM ATP-γ-S and then a solution with high K^+^ (KCl) (Figure 3A). The magnitude of the Ca^2+^ transient evoked by acidification or ATP-γ-S was normalized to the response to high K^+^. A larger number of sensory neurons responded to ATP (92.2%) than extracellular acidification (28.6%) (Figure 3C). About 20.8% of the studied isolated sensory neurons responded to both ATP and extracellular acidification. Genetic deletion of the *Asic3* subunit from sensory neurons did not change the percentage of cells responding to ATP or extracellular acidification. However, the magnitude of the Ca^2+^ transient induced by extracellular acidification was significantly smaller in sensory neurons from conditional *Asic3*-KO mice than controls (Figure 3D). No significant difference in the magnitude of the Ca^2+^ transient triggered by ATP-γ-S was observed between sensory neurons from control and conditional *Asic3*-KO mice. These findings demonstrate that ASIC activation causes an intracellular Ca^2+^ transient in a large proportion of sensory neurons.

Previous studies have shown that ASICs in cortical neurons are permeable to Ca^2+^ [38,39]. Conversely, using pharmacological tools, Boillat and colleagues found that ASIC-induced Ca^2+^ entry in sensory neurons requires VGCCs [40]. To assess whether ASICs and VGCCs are functionally coupled in sensory neurons, we analyzed intracellular Ca^2+^ transients evoked by acidification in the absence and presence of a cocktail of VGCC inhibitors including ω-conotoxin MVIIC (N- and P/Q channel blocker), mibefradil (T-type channel blocker) and nifedipine (L-type channel blocker). It is also well established that ionotropic purinergic receptors (P2xR) are permeable to Ca^2+^ [41]. Accordingly, we found that VGCC blockers do not alter the magnitude of the Ca^2+^ transient evoked by ATP-γ-S in sensory neurons (Figure 3E). In contrast, the presence of VGCC inhibitors in the bathing solution largely suppresses proton-evoked intracellular Ca^2+^ transients (Figure 3E), indicating that native ASICs in sensory neurons do not effectively permeate Ca^2+^. VGCCs, which are activated by depolarization during action potential firing, underlie processes ranging from neurotransmitter release to upregulation of gene expression in neurons [42,43,44]. Our results indicate that the downstream effects of ASICs in sensory neurons are dependent on the action of VGCCs.

### 2.3. Loss of ASIC Function Does Not Alter Bladder Afferent Outflow in Naïve Mice

**Figure 4:**
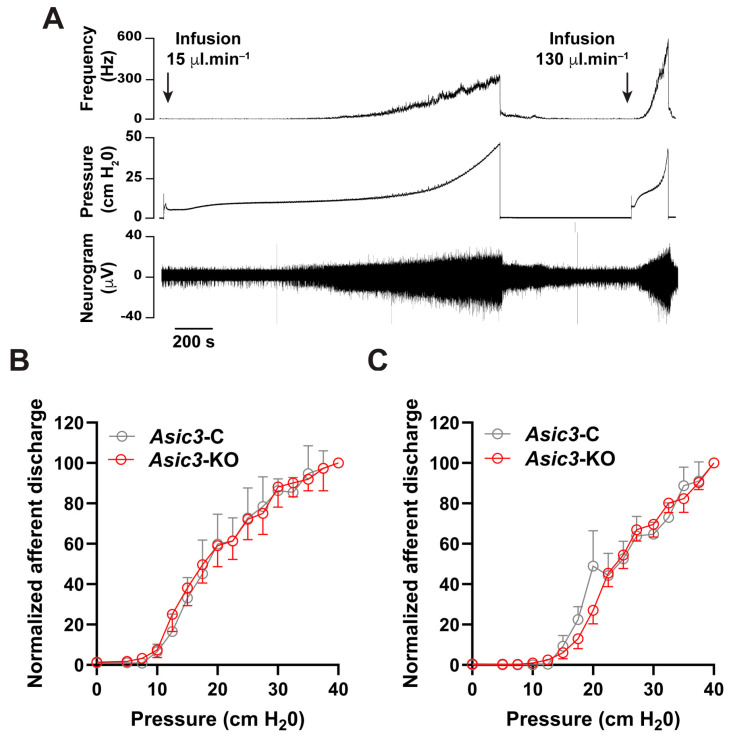
ASICs do not affect afferent discharge in naïve mice. Intravesical pressure and afferent discharge were recorded simultaneously during continuous filling from an ex vivo preparation. (**A**), Representative recording of raw nerve activity, intravesical pressure and afferent discharge for a bladder from a control (*Asic3*-C) mouse is shown. Urinary bladders were distended sequentially at an infusion rate of 15 µL·min^−1^ and 130 µL·min^−1^ until the intravesical pressure reached 40 cmH_2_O. (**B**,**C**), Normalized afferent discharge expressed as a function of the intravesical pressure for bladders infused at 15 µL·min^−1^ (**B**) or 130 µL·min^−1^ (**C**). No difference in bladder afferent discharge was observed between *Asic3*-C and *Asic3*-KO. Data are expressed as the mean ± SEM (*n* = 4–6).

We previously reported that the global genetic deletion of the *Asic3* subunit reduces voiding volume, and the pressure required to trigger micturition [26]. Although *ASIC3* is primarily expressed in sensory neurons [18], *Asic3* mRNA has been detected in other tissues [16,45,46,47,48,49,50,51] including the brainstem [52], an area that contains multiple neural circuits that can profoundly inhibit or facilitate incoming sensory information from the urinary bladder [53]. Therefore, to unambiguously define the function of ASICs in the sensory neurons that innervate the urinary bladder, we measured afferent discharge in response to bladder distension in tissues harvested from naïve control and sensory neuron conditional *Asic3*-KO mice. For these experiments, the bladder, urethra and associated pelvic nerve roots were harvested and mounted in a temperature-regulated chamber that allows recording of single-unit afferent activity during continuous filling [54,55,56]. To record afferent nerve activity, a spinal root (L6 or S1) was carefully positioned in a glass suction electrode and the urinary bladder was continuously filled at a rate of 15 μL·min^−1^. As expected, afferent discharge increased during continuous filling in bladders from both control and conditional *Asic3*-KO mice (Figure 4A,B). No significant difference in afferent discharge/intravesical pressure relationships was noted between the two groups when bladders were filled at a physiological rate of 15 μL·min^−1^ (Figure 4B). This result indicates that ASICs in afferents are not essential for normal bladder function. To determine whether ASICs play a role when bladder wall distension is strained to the limit, we measured afferent discharge during fast filling (130 μL·min^−1^) (Figure 4A,C). Analogously, no significant difference in the afferent discharge/intravesical pressure relationships was seen between *Asic3*-C and conditional *Asic3*-KO mice (Figure 4C). Thus, ASICs do not play a role in the transduction of signals related to distension in naïve mice.

### 2.4. Behavioral Voiding Changes Induced by CYP Are Transient in Control and Conditional Asic3-KO Mice

**Figure 5:**
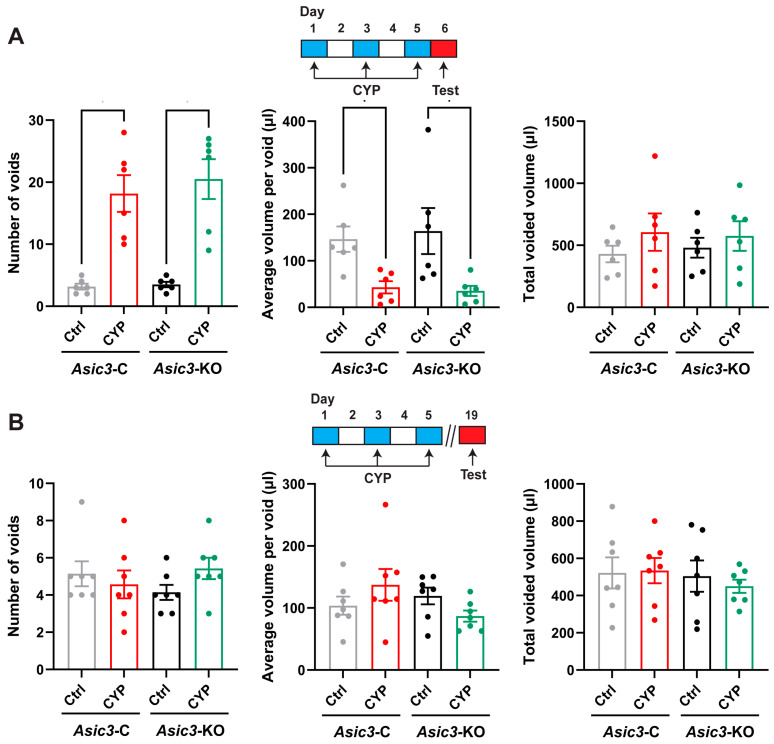
Voiding behavior in freely mobile control and sensory neuron conditional *Asic3*-KO mice. Voiding activity was evaluated over a 6 h time window during the dark phase. Saline (Ctrl) or cyclophosphamide (CYP) were administered every other day for five days. (**A**), Void spot parameters in female control (*Asic3*-C) and conditional *Asic3*-KO mice a day after the final dose of saline or CYP. The protocol of saline or CYP administration, and the evaluation of behavioral changes is shown at the top. (**B**), Void spot parameters in female *Asic3*-C and conditional *Asic3*-KO mice two weeks after the final dose of saline or CYP. The protocol of saline or CYP administration, and the evaluation of behavioral changes is shown at the top. Dots indicate individual data points. Data are shown as mean ± SEM (acute, *Asic3*-C and *Asic3*-KO, *n* = 6; chronic, *Asic3*-C and *Asic3*-KO, *n* = 7). Data were analyzed using the Kruskal–Wallis test followed by Dunn’s multiple comparisons test and significant differences indicated with a single asterisk (*p* ≤ 0.05).

We next explored the contribution that ASIC expressing afferents have in bladder pathophysiology. CYP is an alkylating agent used for chemotherapy for an array of cancers as well as an immune suppressor in nephrotic syndrome, granulomatosis with polyangiitis, and following organ transplantation [57]. CYP is converted to acrolein by microsomal enzymatic hydroxylation in the liver [58]. Cystitis, a common side effect observed in patients that receive CYP [59,60], ensues as acrolein accumulates in the urinary bladder causing pelvic allodynia (i.e., pain due to a stimulus that does not normally provoke pain), frequent voiding events of low volume and inflammation. We have previously shown that *Asic3* null mice are more sensitive to CYP than control mice developing pelvic allodynia with fewer doses of the alkylating agent [27]. *Asic3*-C and sensory neuron conditional *Asic3*-KO mice were injected intraperitoneally with CYP (80 mg/Kg) every other day for five days. To assess conscious voiding behavior in freely moving female mice, we used a recently described video-monitored void-spot assay [61,62,63]. Behavioral testing was conducted a day after receiving the last dose of CYP or saline (acute) and two weeks later (chronic) (Figure 5). Micturition frequency, the volume of individual voiding events and total voided volume were evaluated in a 6 h time window during the mice active dark phase. In the acute setting, both *Asic3*-C and *Asic3*-KO mice treated with CYP exhibit frequent urination with voiding events of smaller volume than mice that received saline (Figure 5A). Interestingly, two weeks after receiving the last dose of CYP, the voiding behavior of *Asic3*-C and conditional *Asic3*-KO mice was indistinguishable from that of mice that received saline (Figure 5B). In sum, conditional deletion of *Asic3* from sensory neurons does not alter voiding behavior. The changes in voiding behavior induced by CYP treatment in both control and sensory neuron conditional *Asic3*-KO mice are transitory and reversible.

### 2.5. ASICs in Afferents Control the Development and Maintenance of Visceral Pain

**Figure 6:**
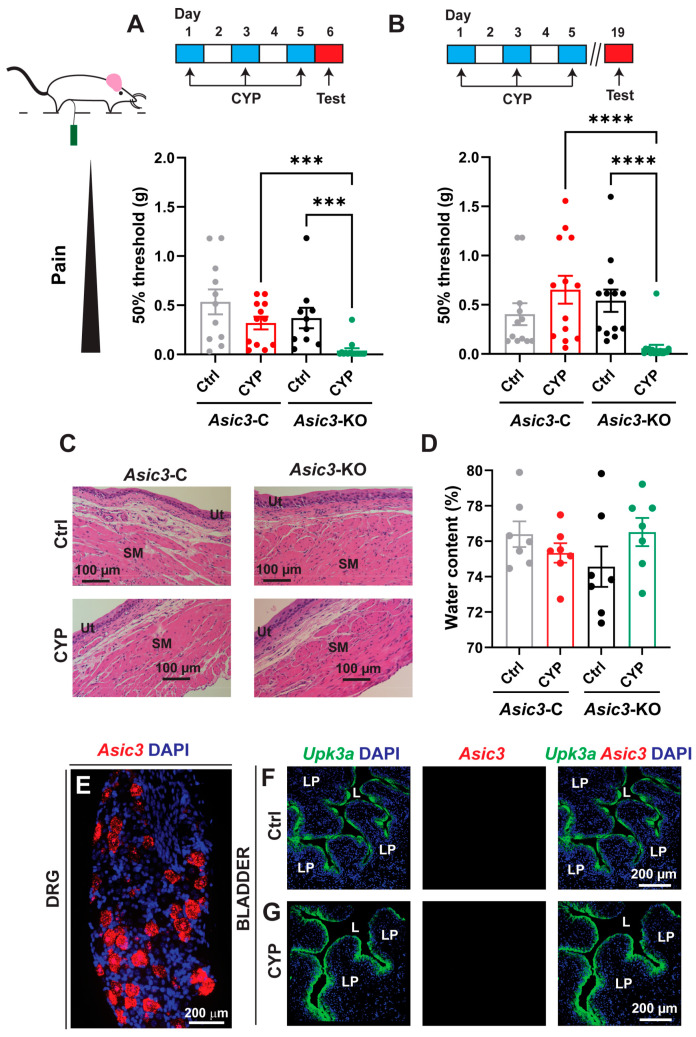
ASIC3 averts pelvic pain in mice exposed to CYP. The protocol for evaluation of behavioral changes following cyclophosphamide (CYP) administration for each condition is shown on the top. (**A**,**B**), 50% withdrawal threshold (g) to von Frey filaments in the pelvic area was evaluated a day (**A**) or two weeks (**B**) after the last dose of saline (Ctrl) or CYP for female control (*Asic3*-C) and sensory neuron conditional *Asic3*-KO mice (*n* = 10–16). Dots indicate individual data points. Data were analyzed using the Kruskal–Wallis test followed by Dunn’s multiple comparisons test and significant differences indicated with a triple asterisk (*p* ≤ 0.001) or a quadruple asterisk (*p* ≤ 0.0001). (**C**), *Asic3*-KO mice treated with CYP do not exhibit signs of bladder inflammation. Bladder sections from *Asic3*-C and *Asic3*-KO mice treated with saline or CYP were stained with hematoxylin and eosin (*n* = 2 for each group). Tissues were harvested two weeks after the last dose of saline or CYP. SM, smooth muscle; Ut, urothelium. (**D**), Tissue water content of urinary bladders from *Asic3*-C and conditional *Asic3*-KO mice treated with saline or CYP (*n* = 7, NS; Kruskal–Wallis test followed by Dunn’s multiple comparisons test). Tissues were harvested two weeks after the last dose of saline or CYP. Dots indicate individual data points. (**E**–**G**), *Asic3* is not expressed in bladder tissues. Confocal images are shown. Fluorescence *in situ* hybridization was performed with probes for *Asic3* and *Upk3a*. Bladder cryosections from wild type mice that received saline (**F**) or CYP (80 mg/Kg) (**G**) every other day for a week. Note that single *Asic3* RNA particles were detected in DRG from wild type mice (**E**), but not in bladder tissues (**F**,**G**). The urothelial marker *Upk3a* is highly expressed in umbrella cells with the lowest expression in the basal cells. LP, lamina propria; L, lumen.

To assess the contribution of ASICs in sensory neurons to bladder nociception, *Asic3*-C and conditional *Asic3*-KO mice were treated with saline (control) or CYP every other day for five days. Somatic sensitivity in the pelvic area was evaluated using von Frey filaments. The 50% withdrawal threshold, which refers to the applied force in the pelvic area by a von Frey filament that elicits a response 50% of the time, was determined with the up–down method described by Chaplan [64] (Figure 6). Note that the lower the 50% withdrawal threshold, the greater the pelvic sensitivity to mechanical stimuli. Consistent with our previous studies [27], sensory neuron conditional *Asic3*-KO mice treated with CYP developed pelvic allodynia with five doses of CYP, while control mice did not exhibit behavioral changes in response to the administration of the alkylating agent (Figure 6A). Interestingly, the pelvic allodynia persisted in conditional sensory neuron *Asic3*-KO mice treated with CYP for at least two weeks (Figure 6B). At this later stage, no apparent pathological features including edema, or the presence of inflammatory cells, were evident in bladder sections stained with hematoxylin and eosin of mice treated with saline or CYP (Figure 6C,D). This finding suggests that persistent visceral pain in conditional *Asic3*-KO mice treated with CYP does not depend on inflammation.

Asic3 expression was reported to increase in the urothelium of rats with chemical cystitis induced by CYP administration [51]. To assess whether *Asic3* expression changes in the bladder wall with CYP treatment, we performed FISH in wild type mice treated with saline or CYP. While we detected message for *Asic3* in sensory neurons of control mice (Figure 1A and Figure 6E), no specific staining was observed in bladder sections of mice injected with saline or CYP (Figure 6F,G). These results demonstrate that Asic3 is primarily expressed in sensory neurons, but not in bladder tissues including the urothelium or detrusor muscle. Thus, ASICs in sensory neurons control the development of visceral pain in mice treated with CYP.

### 2.6. Sensory Neurons Lacking Asic3 Are Prone to Sensitization upon CYP Exposure

**Figure 7:**
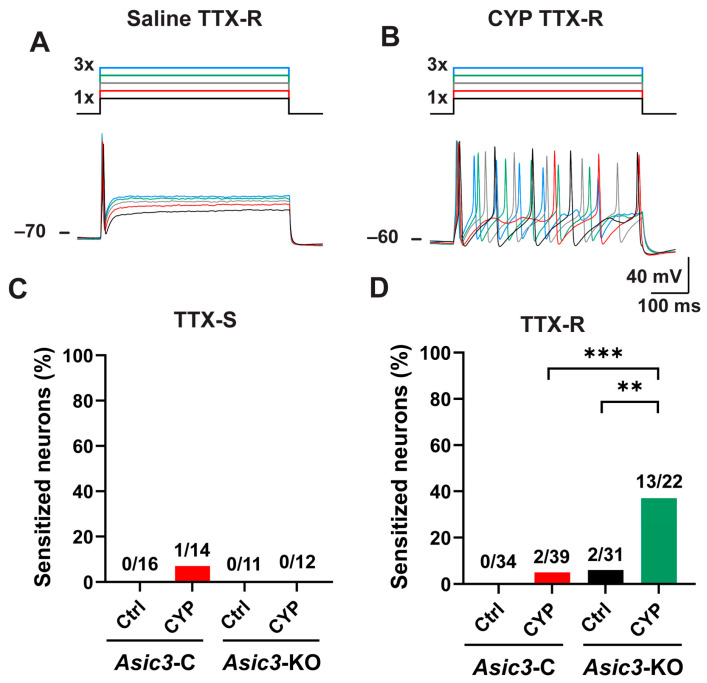
CYP treatment prompts long-term sensitization of a subgroup of bladder sensory neurons in conditional *Asic3*-KO mice. Mice received 3 doses of saline (Ctrl) or cyclophosphamide (CYP). Bladder sensory neurons were isolated from control (*Asic3*-C) and sensory neuron conditional *Asic3*-KO mice two weeks after the last dose of saline or CYP. Firing was evoked by depolarizing currents with a magnitude equal to 1, 1.5, 2, 2.5, and 3 times the rheobase for 500 ms. (**A**) Representative tracing showing the firing in response to electrical stimulation of a tetrodotoxin-resistant (TTX-R) sensory neuron isolated from *Asic3*-KO mice treated with saline. The stimulation protocol is shown on the top. Note that a single action potential was discharged in response to suprathreshold stimuli. (**B**), Representative tracing showing the firing in response to electrical stimulation of a TTX-R sensory neuron isolated from *Asic3*-KO mice treated with CYP. The stimulation protocol is shown on the top. This neuron was deemed sensitized as it discharged multiple action potentials in response to suprathreshold stimuli. (**C**,**D**), TTX-R bladder sensory neurons from conditional *Asic3*-KO mice remain sensitized for weeks after CYP exposure. Percentage of sensitized TTX-sensitive (TTX-S) (**C**) and TTX-R (**D**) bladder sensory neurons. The number of sensitized and non-sensitized neurons for each group and conditions is shown above the bars. Data were analyzed using the two-tailed Chi-square test and significant differences indicated with a double asterisk (*p* ≤ 0.01) or a triple asterisk (*p* ≤ 0.001).

The presence of pelvic pain in conditional *Asic3*-KO mice treated with CYP that persists after the resolution of the inflammation alludes to the sensitization of central nociceptive pathways. Peripheral sensitization, which represents an amplification in the responsiveness of nociceptors that occurs when terminals are exposed to noxious stimuli, in general, requires a pathological condition to be sustained [65]. To investigate whether long-term allodynia in conditional *Asic3*-KO mice treated with CYP is driven, at least in part, by sensitized bladder afferents, we examined the firing of acutely isolated bladder sensory neurons of *Asic3*-C and conditional *Asic3*-KO mice treated with saline or CYP. Neurons were isolated from mice two weeks after the last dose of CYP (or saline) and electrophysiological studies were performed within 2 and 8 h of isolation. Sensory neurons were classified according to the sensitivity of the action potential to tetrodotoxin (TTX), as TTX-resistance (TTX-R) or TTX-sensitive (TTX-S). To assess neuron excitability, we applied an electrical stimulus with an intensity equal to 1, 1.5, 2, 2.5 and 3 times (X) the rheobase (i.e., minimal electrical current required to evoke an action potential) (Figure 7A,B). Most bladder sensory neurons from naïve mice discharge a single action potential in response to suprathreshold stimulation [26]. Thus, neurons that discharge more than an action potential in response to suprathreshold electrical stimulation were considered sensitized. Our studies show that most neurons isolated from *Asic3*-C mice treated with saline or CYP discharged a single action potential in response to suprathreshold stimulation (Figure 7C,D). Interestingly, a significant proportion (13 out of 35 neurons, 37%) of bladder sensory neurons with TTX-R action potentials of conditional *Asic3*-KO mice treated with CYP discharge multiple action potentials upon electrical stimulation (Figure 7D). Taken together, these findings suggest that ASICs control the sensitization of a subgroup of bladder nociceptors.

## 3. Discussion

We demonstrated using genetic and pharmacological tools that ASICs are functionally coupled to VGCCs in sensory neurons. The function of VGCCs is tightly regulated by multiple intracellular factors, including Ca^2+^, which acts as a fast modulator to reduce their activity [66,67]. Most sensory receptors in afferent terminals, including purinergic ionotropic receptors (P2XR), transient receptor potential (TRP) channels and piezo channels are non-selective cation channels permeable to Ca^2+^ [41,68,69]. Thus, activation of these receptors in afferent terminals should suppress VGCC activity. Consistent with this notion, exposure of sensory neurons to ATP evoked an intracellular Ca^2+^ increase that is not blocked by VGCC inhibitors. In contrast, Ca^2+^ transients evoked by extracellular acidification in sensory neurons are inhibited by a cocktail of VGCC blockers. Thus, functional coupling between ASICs and VGCCs perhaps depends on the ability of the former to trigger action potentials without mobilizing Ca^2+^. Many fast and lasting processes that range from membrane repolarization to neurotransmitter release to gene expression are mediated or regulated by intracellular Ca^2+^ dynamics in sensory neurons [70,71]. Thus, multiple possibilities may explain how the activation of VGCCs downstream of ASICs works to avert afferent sensitization and pain; this will need to be examined in future studies.

Analysis of bladder function using continuous cystometry revealed a reduction in the pressure threshold for voiding in global *Asic3* null mice [26]. However, no obvious difference in the mechanical response of bladder afferents to filling was observed between conditional *Asic3*-KO and *Asic3*-C mice. *Asic3* message has been detected in the brainstem [52], where the pontine micturition center (PMC) is located. Upon stimulation, the PMC initiates micturition by promoting detrusor contraction and urethral sphincter relaxation [8,9,10,11,12]. Therefore, the discrepancies between studies with null and conditional *Asic3*-KO suggest a regulatory role for ASICs in central neurons that participate in the micturition circuitry. Taken together, our studies indicate that ASICs in afferents are dispensable for normal bladder mechanotransduction.

On the face of it, conditional deletion of the *Asic3* subunit from sensory neurons recapitulates the regulatory role of ASICs in bladder nociception seen in global null mice [27]. *Asic3*-KO as well as *Asic3*-C develop a pattern of urination with elevated frequency and voids of small volume after CYP treatment, but this voiding behavior resolves. However, pelvic allodynia persists in the absence of inflammation for weeks in conditional *Asic3*-KO mice. The consensus is that sensitized afferents contribute to the acute phase of pain, while central pathways play a major role in the perpetuation of pain in chronic states once the tissue injury has been resolved [65]. The identification of a subgroup of bladder sensory neurons with TTX-R action potentials that remain sensitized weeks after chemical injury was unexpected. We posit that these sensitized sensory neurons contribute to the long-term pelvic allodynia seen in *Asic3*-KO mice treated with CYP. This finding suggests that ASICs work to prevent long-term sensitization in a subset of nociceptors. Many types of nociceptors have been described in the literature [1,3], but future work will be needed to understand their genetic profile and involvement in disease processes.

Multiple subgroups of bladder afferents exist that exhibit unique functional, anatomical and genetic characteristics [72,73,74,75], but their function in physiologic and pathologic processes have remained elusive. Somatic as well as visceral afferents undergo a wide range of molecular changes upon sensitization that alter their function and response to physiological stimuli [1,2,3]. Our studies indicate that the process of sensitization upon CYP exposure is not uniform across bladder afferent subtypes and that sensitization is reversed by different mechanisms in these neuronal populations. This notion is supported by our early studies showing sensitization of bladder sensory neurons with TTX-S action potentials in both control and *Asic3* null mice immediately after CYP treatment [27]. However, we showed here that in the long term the sensitization process of this subset of DRG afferents is reversed. Only a subset of sensory neurons with TTX-R action potentials in conditional *Asic3*-KO mice remains sensitized two weeks after the last dose of CYP. Based on our live cell imaging studies, we posit that the downstream effects of ASICs on nociception are dependent on the action of VGCCs in sensory neurons. In summary, our studies indicate that ASICs play a prominent regulatory role in a subgroup of bladder sensory neurons involved in visceral nociception. Further work is needed to characterize the functional properties and genetic profile of the different types of nociceptors innervating the urinary bladder, their mechanisms of sensitization, and to understand how sensitization is reversed once the tissue injury is resolved.

## 4. Materials and Methods

### 4.1. Reagents

All chemicals were purchased from Sigma-Aldrich (St. Louis, MO, USA), unless otherwise specified.

### 4.2. Mice

Experimental procedures were approved by the University of Pittsburgh Institutional Animal Care and Use Committee. *Asic3^fl/fl^* (*B6;129-Accn3tm2Ccc/Narl*) mice were kindly provided by Dr. Chih-Cheng Chen of the Institute of Biomedical Sciences, Academia Sinica, Taiwan [76]. *Avil*-Cre (*Avil^tm2(cre)Fawa^*), GCaMP5G (*Polr2a^tn(pb-CAG-GaMP5g,tdTomato)Tvrd^*) and C57BL/6J mice were obtained from the Jackson Laboratory (Bar Harbor, ME, USA). Animals were harem bred. *Asic3^fl/fl^* female mice were mated with male *Asic3^fl/–^; Avil-Cre^+/–^* mice to generate sensory neuron conditional *Asic3* knockout (*Asic3*-KO) (*Asic3^fl/fl^*; *Avil-Cre^+/−^*) and control (*Asic3^fl/fl^*) mice. *Asic3^fl/−^*; *Avil-Cre^+/−^*; GCaMP5G^+/−^ were mated with *Asic3^fl/fl^* mice to create conditional *Asic3*-KO and control mice expressing *GCaMP5G* in sensory neurons. Mice were housed in standard cages at the University of Pittsburgh under 12 h light/12 h dark cycles with free access to food and water. The stage of the estrous cycle was not monitored. All experimental mice were 2–6 month-old virgin females and were group housed after weaning at up to 5 per cage. Mice were randomly assigned to control and treated groups. Animals were euthanized by CO_2_ inhalation, followed by a thoracotomy.

### 4.3. Genotyping

Tissue harvested from a tail snip was placed in a 1.5 mL tube and genomic DNA was extracted with QuickExtract DNA Extraction Solution (Cat. N^o^. QE09050, Lucigen, Middlesex, UK). Genotyping for *Asic3*-C and *Asic3*-KO was performed using standard PCR protocols and the following primers: *Asic3^fl/fl^* forward, GATTTGTCACTGCCATGGTG, and *Asic3^fl/fl^* reverse, GGCAGATACTCCTCCTGCT. Genotyping of *Avil*-Cre mice was conducted according to the protocol provided by The Jackson Laboratory using the following primers: *Adv* common, AATGGCTCCCTGTTCACTGT, *Adv* wild type reverse, TGACTAGGTAGAGGTGCAAATGTC, and *Adv* mutant reverse, AGGCAAATTTTGGTGTACGG. Genotyping of GCaMP5G mice was performed according to the protocol provided by The Jackson Laboratory using the following primers: GCaMP5G common, TAGACACATGCCACCAAACC, GCaMP5G wild type reverse, TCTCTCCAGCACCATAACTCC, and GCaMP5G mutant reverse, GATCGATAAAACACATGCGTCA.

### 4.4. Chemical Cystitis

Cyclophosphamide (CYP) (8 mg/mL) in sterile saline was prepared daily and filtered through a sterile 0.45 μm PVDF filter. Mice were injected intraperitoneally with CYP (80 mg/kg) every other day for five days, unless indicated. Control animals received saline. Experimental procedures were carried out either one day or two weeks after the final dose of CYP (or saline). This regimen of CYP administration causes pelvic allodynia and frequent voiding events of small volume [27].

### 4.5. Video-Monitored Void-Spot Assay

Micturition was evaluated in awake and freely moving mice with a video monitoring system described in detail recently [63]. Briefly, the setup consists of a aluminum frame that houses two mouse cages side-by-side, each made of acrylic sheets with a UV-transmitting acrylic bottom (dimensions of 37 × 25 × 20 cm). Mouse cages were equipped with the following: an igloo-shaped sleeping chamber, an Eppendorf tube as a “play toy,” and a dish with standard mouse chow and water in the form of Hydrogel (ClearH_2_O, Westbrook, ME, USA). The bottom of the cages was covered with Cosmo blotting paper (Cat. # 10422-1005; Blick Art Materials, Galesburg, IL, USA). The stand’s lower compartment has reflective mirrored walls and houses two UV tube lights (model T8-F20BLB24 24”, ADJ, Los Angeles, CA, USA), which illuminate evenly the chromatography paper from below. Each mouse was monitored by wide-angle cameras (Logitech C930e, Logitech, San Jose, CA, USA), one positioned above the cage and another mounted at the base of the stand. The mice were housed in a facility with 12-h light–dark cycles, with 7:00 AM being zeitgeber time (ZT) = 0 (start of light cycle). Mice were introduced into the cages between ZT = 9–10 and voiding behavior was evaluated for a 6 h period from ZT = 12–18 (dark cycle). The streamed video was captured with an Apple iMac computer running SecuritySpy version 5 (Ben Software, Kent, UK) at 1 frame per s with 1920 × 1080 pixel resolution. Voiding events were identified by visual inspection of the movies. The void spot area was computed from video images using ImageJ Fiji version 2.3.0 [77] and the void spot volume estimated from a calibration curve generated with known amounts of urine as we described previously [63].

### 4.6. Assessment of Pelvic Mechanical Sensitivity

Mice were acclimatized in modular cages (Bioseb, North Pinellas Park, FL, USA) with a wire-mesh bottom for at least 1 h prior to the test. Withdrawal thresholds to von Frey filaments (Touch Test Sensory Evaluators, North Coast Medical, Morgan Hill, CA, USA) applied to the pelvic area were determined with the up–down method described by Chaplan and colleagues [64]. Briefly, von Frey filaments were applied on the lower abdominal area near the urinary bladder for 1–3 s, with an interval between stimuli of 15 s. Testing was initiated with a filament with a calibrated force of 0.16 g. Abdominal withdrawal (either contraction of the abdominal musculature or postural retraction of the abdomen), licking or scratching in the pelvic area in response to the stimulus were considered a positive response. After the response threshold was first crossed, three additional filaments were applied that varied sequentially up or down based on the animal response. The resulting pattern of positive and negative responses was tabulated, and the 50% withdrawal response threshold was calculated using the equation:

$$50\%\;\mathrm{withdrawal}\;\mathrm{threshold}\;\left(g\right)=\frac{10^{\left[X_{f}+k\delta \right]}}{10000}$$

where *X_f_* represents the value of the final positive von Frey filament used, *k* represents the tabular value for the pattern of positive/negative responses [64], and *δ* represents the mean difference (in log units) between stimuli.

### 4.7. Hematoxylin and Eosin (H&E) Staining

Animals were euthanized by CO_2_ inhalation followed by a thoracotomy, the urinary bladder was harvested, carefully cut open, and pinned mucosal side up onto a rubber sheet submerged in Krebs solution containing (in mM) NaCl 115, NaHCO_3_ 25, KCl 3.8, MgCl_2_ 1, KH_2_PO_4_ 1.2, glucose 10, CaCl_2_ 2.5 buffered at pH 7.4 by gassing with a mixture of 95% O_2_/5% CO_2_ (*v*/*v*). Subsequently, tissues were fixed in 4% (*v*/*v*) paraformaldehyde in phosphate-buffered saline (PBS) for 30 min at 37 °C and then transferred to a 50-mL tube containing 70% ethanol. Each bladder was cut into 3–4 strips, mounted in histology cassettes and then paraffin embedded. Sections (4 µm in thickness) that included all strips were mounted on slides and stained with H&E as previously indicated [62,78]. Images were captured with a Leica DM6000B (Leica, Wetzlar, Germany) upright microscope (fitted with a ×20 HC PL-APO, 0.80 numerical aperture objective) equipped with a Gryphax Prokyon digital camera (Jenoptik, Jena, Germany) and interfaced to an Apple iMac computer running Gryphax software version 2.1.0.724 (Jenoptik, Jena, Germany). The images were exported in TIFF format and compiled in Adobe Illustrator version 28.5 (Adobe, San Jose, CA, USA).

### 4.8. Fluorescence in Situ Hybridization

Expression of *Asic3*, *P2rx3*, *Upk3a* was assessed by fluorescence in situ hybridization (FISH) with the RNAscope^TM^ Multiplex Fluorescent v2 kit (ACD, Newark, CA, USA) and the following probes: Mm-*Asic3* (Cat. N^o^. 480541-C3, ACD, Newark, CA, USA), Mm-*P2rx3* (Cat. N^o^. 521611, ACD, Newark, CA, USA), and Mm-*Upk3a* (Cat. N^o^.505891, ACD, Newark, CA, USA). The 3-plex negative control probe against *Bacillus subtilis dapB* (Cat. N^o^. 320871, ACD, Newark, CA, USA) was used to detect non-specific signals. Bladders and dorsal root ganglia (DRG) were prepared using a fresh-frozen approach. Tissues were dipped into a beaker containing Optimal Cutting Temperature embedding medium (OCT; Tissue-Tek, Sakura Finetek, Torrance, CA, USA), and then transferred to a Tissue-Tek Cryomold (Sakura Finetek, Torrance, CA, USA) filled with OCT. Tissues were frozen by placing the blocks in a −80 °C freezer. Sections (8–12 μm) were cut using a Leica CM1950 cryostat (Wetzlar, Germany) (chamber temperature of −20 °C and a knife temperature of −18 °C), collected on SuperfrostTM Plus microscope slides (ThermoFisher Scientific, Waltham, MA, USA), and “dried” by storing them in the cryostat chamber for 30 min, prior to long-term storage at −80 °C. Slides containing sectioned tissue were removed from the −80 °C freezer, and immediately fixed for 15 min at 4 °C with neutral buffered formalin comprised of 29 mM NaH_2_PO_4_•H_2_O, 45.8 mM Na_2_HPO_4_, and 4.0% (*v*/*v*) paraformaldehyde (neutralized to pH 7.2 while preparing a 40% *w*/*v* stock). Tissue sections were incubated with Protease IV for 5 min at room temperature. Probes were developed using TSA Plus Fluorescein (Akoya Biosciences, Marlborough, MA, USA), TSA Plus Cyanine (Akoya Biosciences, Marlborough, MA, USA). Slides incubated with probes for *Asic3*, *P2rX3*, *Upk3a* and negative control probes were run side-by-side and treated identically. Labeled tissues were mounted using ProLong Gold antifade mounting (Cat. # P36934, ThermoFisher Scientific, Waltham, MA, USA) and cured overnight at room temperature in the dark. The slides were then stored at 4 °C. General image capture was performed using a Leica HCX PL APO 20× (Wetzlar, Germany), 0.75 numerical aperture dry objective and the appropriate laser lines of a Leica Microsystems SP8 Stellaris (Wetzlar, Germany) confocal microscope outfitted with a 405-laser diode and a white light laser. 8-bit images were collected at 600 Hz using 2-line averages combined with 2 frame averages. Cross-talk between channels was prevented by use of spectral detection coupled with sequential scanning. Stacks of images (1024 × 1024, 8-bit) were collected using system-optimized parameters for the z-axis. Images were processed using the 3D visualization package of Leica LASX software version v4.6.1.27508 (Wetzlar, Germany) and exported as TIFF files. Final figures were assembled in Adobe Illustrator version 28.5 (Adobe, San Jose, CA, USA).

### 4.9. Tissue Edema Quantification

To assess edema in urinary bladders of mice treated with saline or CYP, tissues were harvested, carefully dried with filter paper and weighed to obtain the wet mass (WM). To determine the dry mass (DM), samples were placed in an incubator at 55 °C until constant weight was reached (typically after 5 days). The percentage of water tissue content was calculated using the following equation:

$$\%\;\mathrm{water}\;\mathrm{content}=\frac{\mathrm{WM}-\mathrm{DM}}{\mathrm{WM}}\text{×}100$$

### 4.10. Retrograde Labeling of Bladder Sensory Neurons

Bladder afferent neurons were labeled with the fluorescent retrograde axonal tracer DiI (1,1′-dioctadecyl-3,3,3′,3′-Tetramethylindocarbocyanine perchlorate (Cat. N^o^. D282, ThermoFisher Scientific, Waltham, MA, USA) as previously described [26,27]. Mice were anesthetized with isoflurane and an abdominal incision was made to expose the urinary bladder. Dil (5% *w*/*v* in DMSO) was injected at three to four locations in the bladder wall. Then, the abdominal incision was closed in layers using 5.0 PDO absorbable monofilament surgical sutures (AD Surgical, Sunnyvale, CA, USA). Ketoprofen (5 mg/kg) was administered subcutaneously to alleviate pain (Zoetis, Parsippany, NJ, USA) and ampicillin (100 mg/kg) (Eugia, Hightstown, NJ, USA) to prevent infections. DRG were harvested between 7 and 10 days after the surgical procedure.

### 4.11. Isolation of Bladder Lumbosacral Sensory Neurons

Dorsal root ganglia (DRG) from lumbar 6 to sacral 2 were collected from 2–3 mice injected with Dil and placed in a cell culture dish containing neurobasal-A medium (Cat. N^o^. 10888022, ThermoFisher Scientific, Waltham, MA, USA). DRG were cut into small fragments and then incubated in a flask with 5 mL of neurobasal-A medium supplemented with 10 mg of collagenase type 4 (Cat. N^o^. LS004188, Worthington Biochemical Corporation, Lakewood, NJ, USA) and 5 mg of trypsin (Cat. N^o^. LS003703, Worthington Biochemical Corporation, Lakewood, NJ, USA) for 30 min at 37 °C. Tissue fragments were carefully triturated with a fire-polished glass pipette, and the cell suspension was centrifuged at 460× *g* for 5 min. The pellet, containing DRG somas, was resuspended in complete neuro media (neurobasal-A media supplemented with 5% of B27 supplement, 0.5 mM L-glutamine, 100 U/mL of penicillin and 100 μg/mL of streptomycin). This washing step was repeated three additional times. The final pellet was resuspended in 1.5 mL of complete neuro media and the suspension was plated on coverslips coated with ornithine (Cat. N^o^. P4957, Sigma, St. Louis, MO, USA) and laminin (Cat. N^o^. 23017-015, ThermoFisher Scientific, Waltham, MA, USA) inside a 6 well plate. After 2 h at 37 °C, warm complete neuro media was added to the wells. For long term studies, complete neuro media was supplemented with 100 ng/mL of nerve growth factor (Cat. N^o^. 13290-010, ThermoFisher Scientific, Waltham, MA, USA).

### 4.12. Electrophysiology

Patch-clamp studies were conducted between 2–8 h after plating the DRG preparation on glass coverslips, unless otherwise indicated. Recordings were performed in a chamber mounted on the stage of a Nikon Ti inverted microscope suited with a Sedat Quad set (Chroma Technology, Bellows Falls, VT, USA), a Lambda XL light source (Sutter Instruments, Novato, CA, USA), and an ORCA-Flash 2.8 camera (Hamamatsu, Bridgewater, NJ, USA). Dil-labeled neurons were identified with an ET555/25x excitation filter (Chroma Technology, Bellows Falls, VT, USA). Electrophysiological recordings were performed at room temperature with a PC-505B patch-clamp amplifier (Warner Instruments, Holliston, MA, USA) interfaced to a Digidata 1440A (Molecular Devices, San Jose, CA, USA) acquisition system. Data were acquired with a PC computer running pClamp 10.7 (Molecular Devices, San Jose, CA, USA). Electrical signals were low-pass filtered at 1 kHz (four-pole Bessel filter) and digitized at 5 kHz. Micropipettes with a fire-polished tip and a resistance of 1.5–3 mΩ were used for whole-cell recordings. The pipette filling solution contained the following (in mM): 145 KCl, 1 MgCl_2_, 0.1 CaCl_2_, 1 EGTA, and 10 HEPES (pH 7.2). The extracellular bath solution contained the following (in mM): 135 NaCl, 5 KCl, 1 MgCl_2_, 2.5 CaCl_2_, 10 glucose and 10 HEPES (pH 7.4 or 8) or MES (pH 6.0 or 6.5). Whole-cell access was gained with amphotericin B (120 μg/mL). Rapid fluid delivery was supplied by a perfusion pencil positioned near the cell. Proton- and ATP-evoked currents were recorded at a membrane potential of −60 mV, unless otherwise indicated. For current-clamp studies, only cells with a resting membrane potential more negative than −40 mV and that generated action potentials with a distinct overshoot exceeding 0 mV in response to a depolarizing current were included in the study. To analyze the repolarization phase of action potentials evoked by extracellular acidification or ATP (used as ATP-γ-S, a slowly hydrolysable ATP analog), the first mathematical derivative of the membrane potential was calculated.

Neuronal excitability was evaluated in responses to suprathreshold current pulses, as we previously described [26,27,62,79,80,81]. Briefly, the action potential rheobase (i.e., minimum current required to elicit an action potential) was determined by applying a sequence of 4 ms rectangular depolarizing current pulses of increasing intensity at 1 s intervals. Then, neurons were stimulated with current pulses with a duration of 500 ms and intensities of 1, 1.5, 2, 2.5, and 3 times the rheobase at 4 s intervals. At the end of each experiment, neurons were exposed to tetrodotoxin (TTX) and classified based on the sensitivity of the action potential to the toxin as tetrodotoxin-sensitive (TTX-S) or TTX-resistant (TTX-R). Most bladder sensory neurons from naïve mice discharge a single action potential in response to electrical stimulation [26,27,62,79,80,81]. Therefore, neurons that discharged more than one spike in response to electrical stimulation were considered sensitized. Accordingly, the percentage of sensitized TTX-R and TTX-S neurons was estimated for each experimental group.

### 4.13. Intracellular Ca^2+^ Imaging

L6-S2 DRG neurons were isolated from naïve *Avil*-Cre^+/−^; GCaMP5G^+/–^ or *Asic3^fl/fl^*; *Avil*-Cre^+/−^; GCaMP5G^+/−^ mice and plated on coverslips as described above. Experiments were conducted 24–72 h after plating. Coverslips with sensory neurons were placed in a recording chamber (volume 300 μL) with heating elements and continuously perfused with recording solution containing (in mM) 135 NaCl, 5.0 KCl, 1 MgCl_2_, 2.5 CaCl_2_, 10 glucose, 10 HEPES, pH 7.4. Acidic test solutions of pH 5 were buffered with MES. The high K^+^ solution (KCl) contained (in mM): 40 NaCl, 100 KCl, 1 MgSO_4_, 2 CaCl_2_, 10 glucose, 10 HEPES, pH 7.4. The temperature of the chamber and solutions was maintained at ~37 °C with a dual channel bipolar temperature controller model TC-344B (Warner Instruments, Holliston, MA, USA). Ca^2+^ imaging was performed with an upright Olympus microscope model BX51W suited with a water immersion 20× UMPlanFL N objective (Olympus, Center Valley, PA, USA), a Hamamatsu CMOS ORCA fusion camera (Hamamatsu Corporation, Bridgewater, NJ, USA), and a Lambda XL light source (Sutter Instruments, Novato, CA, USA). Images were acquired and analyzed with cellSens software 3.4 (Olympus, Tokyo, Japan). The perfusion system (Automate Scientific, Berkeley, CA, USA) was remotely operated through a Digidata 1440A (Molecular Devices, San Jose, CA, USA). Images were acquired every 160 ms. The change in fluorescence intensity is denoted as ΔF/F, where F is the fluorescence intensity of GCaMP5G at time 0 and ΔF is the difference between the fluorescence intensity maxima and the basal at time 0. Data represent means ± SEM (*n*), where *n* equals the number of cells analyzed from at least two DRG preparations for each condition.

### 4.14. Ex Vivo Afferent Nerve Recordings

Mice were euthanized by CO_2_ inhalation followed by thoracotomy and exsanguination. The bladder, urethra and associated L6-S1 spinal nerve roots were dissected in a cold Krebs solution consisting of the following (in mM): NaCl 118.5, KCl 4.7, NaHCO_3_ 25, NaH_2_PO_4_ 1.3, MgSO_4_ 1.2, D-Glucose 18, and CaCl_2_ 2.5. A cannula was inserted in the urethra and the ureters were tied close to the urinary wall. The urinary bladder and associated tissues were placed in a recording chamber perfused with warm Krebs solution bubbled with a mixture of 95% O_2_/5% CO_2_ (*v*/*v*). The temperature of the chamber was maintained at 35 °C with a dual temperature controller (TC-344B; Warner instruments, Holliston, MA, USA). The urethral cannula was coupled to a four-way connector as follows: one branch led to a pressure transducer (TBM4M 4-Channel Transducer Amplifier, WPI, Sarasota, FL, USA), a second port was connected to a syringe pump (SP100iZ, WPI, Sarasota, FL, USA) for continuous infusion with Krebs, and the third port to a cannula with a pressure release valve, which facilitated bladder emptying after filling. To record afferent nerve activity induced by bladder distention, a spinal root (L6 or S1) was carefully positioned in a glass suction electrode. Electrical signals were recorded with a differential amplifier Model 1700 (A-M Systems, Sequim, WA, USA) and band-pass filtered at 100–1000 Hz. The amplified signal was passed through a Hum Bug Noise Eliminator (Digitimer, Hertfordshire, UK) to remove 50/60 Hz noise and harmonics. Pressure and nerve signals were recorded at a rate of 25 kHz with a CED 1401 Power 3A data acquisition system (Cambridge Electronic Design, Cambridge, UK) interfaced to a PC computer running Spike 2 software version 10.19 (Cambridge Electronic Design, Cambridge, UK).

The preparation was equilibrated in the chamber for at least 30 min. To assess the viability of the preparation and reproducibility of the afferent response to bladder filling, urinary bladders were continuously filled at a rate of 15 μL·min^−1^ to a pressure of 40 cmH_2_O. Once this pressure was reached, the bladder was emptied through the urethral cannula by manually opening the release valve. This stimulation was repeated two additional times with a resting interval of 10 min. Finally, urinary bladders were continuously filled at 15 or 130 mL·min^−1^. A maximum bladder pressure of 40 cmH_2_O (29 mmHg) was chosen because it reflects the peak pressure achieved at micturition during cystometrograms in mice [26,82]. The threshold for detection of afferent nerve activity was set at twice the root mean square (RMS) of the electrical signal when the bladder was empty. Action potentials were detected with Spike 2 software version 10.19 (Cambridge Electronic Design, Cambridge, UK). The frequency of firing at a given pressure was calculated for a 5 s or 0.58 s interval for bladder distended at 15 or 130 mL/min, respectively.

### 4.15. Statistical Analysis

Data are expressed as mean ± SEM (*n*), where *n* equals the number of independent measurements. Parametric or nonparametric tests were employed as appropriate. *p* < 0.05 was considered statistically significant. Statistical comparisons were performed with GraphPad Prism 9 (GraphPad Software, San Diego, CA, USA).

## Data Availability

The raw data supporting the conclusions of this article will be made available by the authors on request.
